# Cryptococcal antigenemia is associated with meningitis or death in HIV-infected adults with CD4 100–200 cells/mm^3^

**DOI:** 10.1186/s12879-020-4798-1

**Published:** 2020-01-20

**Authors:** James Wykowski, Sean R. Galagan, Sabina Govere, Carole L. Wallis, Mahomed-Yunus Moosa, Connie Celum, Paul K. Drain

**Affiliations:** 1 Department of Medicine, University of Washington, Seattle, USA; 2 Global Health, University of Washington, Seattle, USA; 3 AIDS Healthcare Foundation, Durban, South Africa; 4 BARC-SA and Lancet Laboratory, Johannesburg, South Africa; 5 Department of Infectious Diseases, University of KwaZulu-Natal, Durban, South Africa; 6 Epidemiology, University of Washington, Seattle, USA

**Keywords:** HIV, Cryptococcal meningitis, Cryptococcal antigen, Screening, Opportunistic infections, Sub-Saharan Africa

## Abstract

**Background:**

Cryptococcal antigen (CrAg) screening with fluconazole prophylaxis has been shown to prevent cryptococcal meningitis and mortality for people living with HIV (PLWH) with CD4 < 100 cells/mm^3^. While cryptococcal meningitis occurs in individuals with CD4 100–200 cells/mm^3^, there is limited evidence that CrAg screening predicts cryptococcal meningitis or mortality among this group with moderate immunosuppression. Current IDSA and WHO clinical guidelines recommend restricting CrAg screening to PLWH with CD4 < 100 cells/mm^3^.

**Methods:**

We conducted a prospective cohort study of PLWH 18+ years who had not initiated ART in South Africa. We followed participants for 14 months to determine onset of cryptococcal meningitis or all-cause mortality. At study completion, we retrospectively tested stored serum samples for CrAg using an enzyme immunoassay (EIA). We calculated CD4-stratified incidence rates of outcomes and used Cox proportional hazards to measure associations between CrAg positivity and outcomes.

**Results:**

We enrolled 2383 PLWH, and 1309 participants had serum samples tested by CrAg EIA. The median CD4 was 317 cells/mm^3^ (interquartile range: 173–491 cells/mm^3^). By CD4 count at baseline, there were 209 individuals with a CD4 count of 100–200 cells/mm^3^ and available CrAg test results. Of these, four (1.9%) tested positive. Two of four (IR: 58.8 per 100 person-years) CrAg+ participants and 11 of 205 (IR: 5.6 per 100 person-years) CrAg- participants developed cryptococcal meningitis or died for an overall rate of death or cryptococcal meningitis that was 10.0-times higher for those who were CrAg+ (95% confidence interval: 2.2–45.3). Among those with CD4 < 100 cell/mm^3^ and CrAg EIA test results (*N* = 179), ten (5.6%) participants tested CrAg+. Among this group, seven of ten (IR: 137.6 per 100 person-years) CrAg+ participants and 26 of 169 (IR: 17.8 per 100 person-years) CrAg- participants developed cryptococcal meningitis or died, for a rate of death or cryptococcal meningitis that was 6.3-times higher for those who were CrAg+ (95% confidence interval: 2.7–14.6).

**Conclusions:**

Although few PLWH with moderate immunosuppression screened CrAg positive, a positive CrAg test was predictive of increased risk of cryptococcal meningitis or death. Similar to those with a CD4 < 100 cell/mm^3^, systematic CrAg screening may reduce morbidity and mortality in PLWH with CD4 100–200 cells/mm^3^.

## Background

Despite a global increase in access to antiretroviral therapy (ART), cryptococcal meningitis continues to be a significant cause of morbidity and mortality among people living with HIV (PLWH) [1]. The majority of cryptococcal meningitis cases occur in low- and middle-income countries, particularly in sub-Saharan Africa [2]. Serum screening for cryptococcal antigen (CrAg) and subsequent prophylaxis for those who screen positive limits the burden of disease by preventing the progression from cryptococcal antigenemia to cryptococcal meningitis or death [1, 3]. Asymptomatic cryptococcal antigenemia is also associated with early all-cause mortality in PLWH in sub-Saharan Africa [4].

According to current World Health Organization (WHO) and Infectious Disease Society of America (IDSA) guidelines, screening for CrAg is reserved for PLWH who have a CD4 count less than 100 cells/mm^3^ [1, 5]. However, the WHO also states that screening may be considered at a higher CD4 threshold of < 200 cells/mm^3^, without specifying the specific circumstances in which to increase the threshold for CrAg screening [1].

South Africa is one of many countries in sub-Saharan Africa that now incorporates CrAg screening into its national HIV guidelines for people with CD4 < 100 cells/mm^3^ [6]. A previous meta-analysis established a pooled CrAg positivity prevalence of 2.0% for PLWH with CD4 100–200 cells/mm^3^ [7], and recent data from sub-Saharan Africa suggests that at least 9% of cases of cryptococcal meningitis present in individuals with CD4 > 100 cells/mm^3^ [8, 9]. We sought to characterize the association of CrAg positivity by the standard of care serum enzyme immunoassay (EIA) with cryptococcal meningitis or death among PLWH with CD4 100–200 cells/mm^3^.

## Methods

### Study design

We enrolled adults seeking HIV testing who presented to the iThembalabantu People’s Hope Clinic, which provides free HIV care for over 10,000 residents of the Umlazi township in KwaZulu-Natal, South Africa. The study included participants who were ART-naïve, newly diagnosed with HIV, English or Zulu speaking, and older than 17 years of age. The full design of the parent study has been previously described [10]. We excluded participants who were pregnant as well as participants who had received antifungal therapy in the previous three months. We obtained study approval by the University of Washington’s Institutional Review Board and the University of KwaZulu-Natal’s Medical Research Ethics Committee.

### Data collection

At the time of enrollment, participants received rapid HIV testing. HIV-infected participants met with a research nurse, who obtained serum samples and completed a medical history and symptom questionnaire. Blood samples were obtained, CD4 count was tested using *FACSCalibur* flow cytometry at an offsite laboratory, and serum samples were stored. HIV testing and follow up treatment was in accordance with South African ART treatment guidelines [11]. Participants also underwent the WHO 4-symptom screen for tuberculosis (TB). Upon the completion of a 12-month follow-up period, we retrospectively tested stored serum samples for CrAg using the ALPHA CrAg EIA test system developed by Immy Diagnostics (Norman, Oklahoma, USA).

### Outcomes

We followed participants for 12 months following HIV diagnosis, and collected data at regular intervals. Our primary outcomes of interest were all-cause mortality, cryptococcal meningitis, and a combined outcome of mortality or cryptococcal meningitis. For participants lost to follow-up, we searched the national South African death registry to obtain mortality outcomes. Outcomes that occurred up to 14 months following enrollment were included and any outcomes ascertained after 14 months were excluded from the analysis.

### Data analysis

We calculated the incidence of study outcomes and assessed the relationship between serum CrAg positivity (positive compared to negative or indeterminate) and outcomes using a time-to-events analysis (Cox proportional-hazards regression and Kaplan-Meier). Incidence rates (IR) were calculated as the number of outcomes divided by the person-time at risk (person-years) up to 14 months after enrollment, accounting for loss to follow-up. Analyses were stratified by CD4 count at enrollment (< 100 or 100–200 cells/mm^3^).

## Results

In total, we enrolled 2383 adults from September 2013 to November 2017 (Table 1). The majority (57.6%) of participants were female, and the mean age was 33.1 years (standard deviation: 9.3 years). 54.6% of participants did not complete high school, and 43% of participants were employed. Of the 2360 participants who reported income data, 75.6% had a monthly income of less than 2000 South African rand per month (<USD $150/month).

**Table 1:** Baseline characteristics of study participants (*N* = 2383)

	N (%)
Sociodemographics	
Age (years): mean (SD)	33·1 (9·3)
Female gender	1372 (57·6)
Education	
None, primary or some high school	1300 (54·6)
Completed high school or higher degree	1079 (45·4)
Marital status	
Married	150 (6·3)
Single (never married)	2211 (92·8)
Widowed/Divorced	22 (0·9)
Number of children (*N* = 2368)	
No children	448 (18·9)
1 child	795 (33·6)
≥ 2 children	1125 (47·5)
Employed	1026 (43·0)
Income level (South African rand/month) (*N* = 2360)	
< 2000 rand (< USD $150)	1785 (75·6)
> 2000 rand (> USD $150)	575 (24·4)
HIV and Medical History	
Previously tested HIV	1705 (71·7)
Previously tested HIV positive, among those tested (*N* = 1694)	430 (25·4)
Partner HIV status (*N* = 2374)	
Unknown	1315 (55·4)
HIV-negative	390 (16·4)
HIV-positive	669 (28·2)
Ever tested *Cryptococcus* positive	5 (0·2)
Ever received *Cryptococcus* treatment, among those tested positive (*N* = 5)	3 (60·0)
Clinical and Laboratory Testing	
CD4 count (cells/mm^3^): median (IQR)	317 (173, 491)
< 100 cells/mm^3^	325 (13·6)
100–199 cells/mm^3^	354 (14·9)
200+ cells/mm^3^	1656 (69·5)
Not available	48 (2·0)
Laboratory-based serum Cryptococcal Antigen EIA (*N* = 1308)	
Positive	15 (1·1)
Negative	1294 (98·8)

EIA—enzyme immunoassay; POC—point-of-care

The median CD4 count was 317 cells/mm^3^ (interquartile range: 173–491 cells/mm^3^). Three hundred and twenty-five participants (13.6%) had CD4 < 100 cells/mm^3^, 354 (14.9%) participants had CD4 100–200 cells/mm^3^, and 1656 participants (69.5%) had CD4 > 200 cells/mm^3^. CD4 data were not available for 48 participants. Five participants had previously tested positive for CrAg, and three of these five participants previously received treatment with fluconazole.

At enrollment, participants frequently reported symptoms associated with cryptococcal meningitis, including fatigue (43.4%), headache for greater than 24 h (25.5%), fever (24.5%), neck stiffness (16.2%), difficulty walking (13.1%), vision changes (9.3%), and seizure within the last seven days (1.0%). Thirty-six participants (1.5%) had received fluconazole preventative therapy at one-year follow-up.

Serum EIA testing was only available from November 2013 to August 2016, and thus not all participants were able to receive serum EIA testing. In total, 1309 participants (54.9%) received EIA testing, and of those 15 (1.1%) were CrAg positive. Participants who did not receive EIA testing still received other point-of-care CrAg screening in accordance with the larger study protocol. There were no statistically significant differences in age, gender, or CD4 count between the group that received EIA testing and the group that did not. CrAg testing was positive in one participant with CD4 > 200 cells/mm^3^, four participants with CD4 100–200 cells/mm^3^, and ten participants with CD4 < 100 cells/mm^3^.

**Fig. 1:**
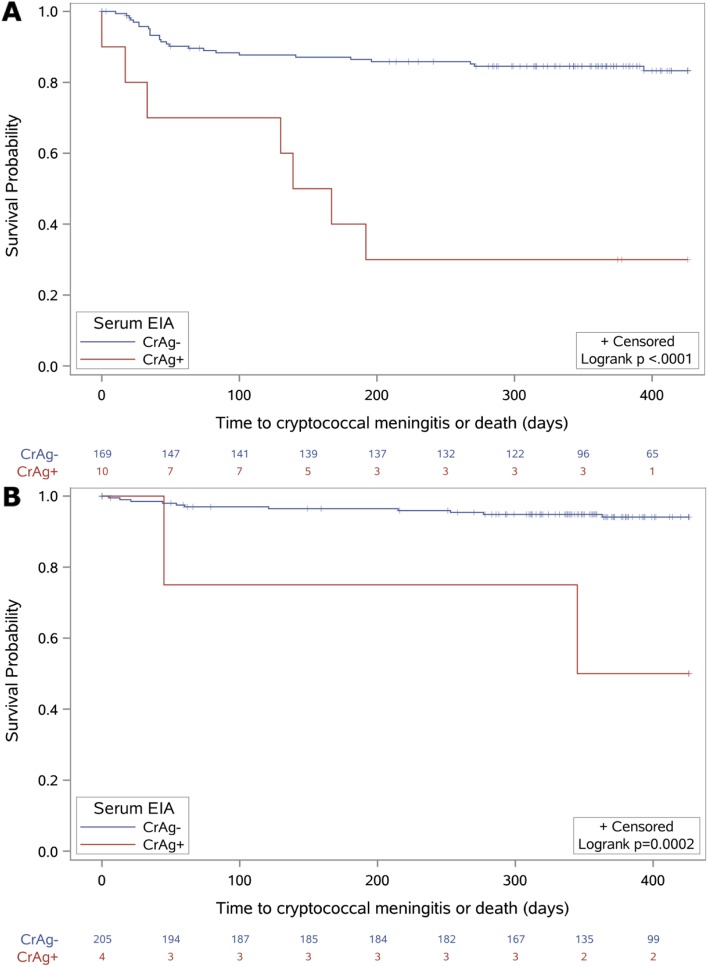
Time to cryptococcal meningitis or death by laboratory-based serum EIA CrAg test result, among patients with a baseline (**a**) CD4 count< 100 or (**b**) 100–200 cells/mm^3^

Among participants with CD4 < 100 cells/mm^3^ (*N* = 179), four of ten (IR: 64.7 per 100 person-years) CrAg positive participants and 26 of 169 (IR: 19.7 per 100 person-years) CrAg negative participants died, and four of ten (IR: 78.6 per 100 person-years) CrAg positive participants and 1 of 169 (IR: 0.7 per 100 person-years) CrAg negative participants developed cryptococcal meningitis. In aggregate, seven of ten (IR: 137.6 per 100 person-years) CrAg positive participants and 26 of 169 (IR: 17.8 per 100 person-years) CrAg negative participants died or developed cryptococcal meningitis (Fig. 1). In this group, CrAg positivity was strongly associated with increased risk of meningitis or death (HR: 6.3, 95% confidence interval [CI]: 2.7–14.6, *p* < 0.001) (Table 2).

**Table 2:** Incidence of cryptococcal meningitis or death within 14 months of enrollment by laboratory-based EIA testing among participants with low CD4 count at enrollment

				*Incidence Rate*^*1*^	*Unadjusted*	
*CD4 count at enrollment*	*N*	*Events (n)*	*py*		*HR (CI)*	*p=*
100–200 cells/mm^3^						
CrAg positive	4	2	3.4	58.8	10.0 (2.2, 45.3)	0.003
CrAg negative or indeterminate	205	11	196.2	5.6	Ref	
<100 cells/mm^3^						
CrAg positive	10	7	5.1	137.6	6.3 (2.7, 14.6)	< 0.001
CrAg negative or indeterminate	169	26	145.8	17.8	Ref	

*CI* 95% confidence interval, *CrAg Cryptococcus* antigen, *EIA* enzyme immunoassay, *HR* Hazard ratio, *py* person-years

^1^# of total outcomes per 100 person-years

In those with CD4 100–200 cells/mm^3^ (*N* = 209), two of four (IR: 58.8 per 100 person-years) CrAg positive participants and 11 of 205 (IR: 5.6 per 100 person-years) CrAg negative participants died, and none developed cryptococcal meningitis. In aggregate, two of four (IR: 58.8 per 100 person-years) CrAg positive participants and 11 of 205 (IR: 5.6 per 100 person-years) CrAg negative participants died or developed cryptococcal meningitis. CrAg positivity was associated with an increased hazard of death or cryptococcal meningitis (HR 10.0, CI: 2.2–45.3, *p* = 0.003).

## Discussion

In this cohort of PLWH in sub-Saharan Africa, serum CrAg positivity was associated with increased risk of death or cryptococcal meningitis among participants with CD4 100–200 cells/mm^3^ as well as those with CD4 < 100 cells/mm^3^. The prevalence of CrAg positivity among those with CD4 100–200 cells/mm^3^ was 1.9%, which is consistent with prior studies [7]. There was one participant who was CrAg positive who had CD4 > 200 cells/mm^3^. This participant did not die or develop cryptococcal meningitis.

Our findings build on previous studies, which have individually reported the prevalence of CrAg and cryptococcal meningitis or death for PLWH with CD4 100–200 cells/mm^3^, but not their association. One study located in Uganda previously found that 9% of cryptococcal meningitis cases in PLWH presented in patients with a CD4 > 100 cells/mm^3^ [9], and another study from South Africa found a prevalence of 12.5% in this group [8].

While PLWH with CD4 < 100 cells/mm^3^ who are CrAg positive are most likely to have adverse outcomes, our results suggest CrAg screening may be beneficial for PLWH who have CD4 100–200 cells/mm^3^. Many participants reported symptoms suggestive of cryptococcal meningitis, such as neck stiffness, vision changes, and persistent headache; testing for serum CrAg could better characterize the pre-test probability of cryptococcal meningitis in patients presenting with relatively non-specific symptoms. Given that we only identified one CrAg positive participant with CD4 > 200 cells/mm^3^ who did not die or develop cryptococcal meningitis, we did not find any evidence to support raising the screening threshold to CD4 > 200 cells/mm^3^ in this setting.

Laboratory-based lateral flow assay (LFA) CrAg screening for PLWH with CD4 < 100 cells/mm^3^ has been shown to be life-saving and cost-effective when implemented in Uganda and South Africa [12, 13]. Despite a relatively low CrAg prevalence in PLWH with CD4 100–200 cells/mm3, using a low-cost screening test and prophylactic fluconazole would likely be cost-effective when compared to the high costs of cryptococcal meningitis and/or mortality. A sensitivity analysis of CrAg screening in PLWH with CD4 < 100 cells/mm^3^ demonstrated that CrAg screening was cost-effective as long as the CrAg prevalence remained greater than .5% [13]. Future studies should seek to evaluate the effect of fluconazole therapy in PLWH with CD4 100–200 cells/mm3, and model the costs associated with raising the CrAg screening threshold.

Our study took place in South Africa, a country with a high incidence of meningitis [6]. This likely affected the yield of screening in our cohort. Increasing the threshold for CrAg screening to 200 cells/mm^3^ may be less appropriate in regions with a lower incidence of cryptococcal meningitis, but more research is needed in different geographical areas.

Our study is limited by the small number of CrAg positive participants and person-time in which to assess outcomes. We used a combined outcome of death or confirmed cryptococcal meningitis in our study, although we suspect many of the deaths identified through searching the national registry could have been due to death from cryptococcal meningitis despite the participants not presenting for further evaluation. PLWH are at increased risk of multiple opportunistic infections, not just cryptococcal meningitis. In particular, KwaZulu-Natal has a high rate of TB and HIV/TB co-infection [14]. While we did assess TB symptoms at the time of enrollment, we were unable to reliably assess for development of TB-related mortality. Overall, our study findings are limited by the inability to confirm cause of death in all participants. In selecting the gold standard for our study, we opted to use the serum EIA test based on its proven accuracy for CrAg screening.

## Conclusions

In the subset of newly identified PLWH with a CD4 100–200 cells/mm^3^ in South Africa, serum CrAg positivity was observed in almost 2% of participants, and a positive CrAg result was strongly associated with an increased risk of cryptococcal meningitis or death during a 12-month follow-up period. Raising the threshold for CrAg screening to 200 cells/mm^3^ could help reduce morbidity and mortality in PLWH in cryptococcus-endemic regions.

## Data Availability

The datasets used and analyzed during the current study are available from the corresponding author on reasonable request.

## Abbreviations

ART: Antiretroviral Therapy
CI: Confidence Interval
CrAg: Cryptococcal antigen
EIA: Enzyme Immunoassay
HIV: Human immunodeficiency virus
HR: Hazard Ratio
IDSA: Infectious Diseases Society of America
IR: Incidence Rate
LFA: Lateral flow assay
PLWH: People living with HIV
TB: Tuberculosis
USD: United States Dollar
WHO: World Health Organization
